# A Systematic Review on the Role of Surgical Drains in Vascular Surgery

**DOI:** 10.7759/cureus.83941

**Published:** 2025-05-12

**Authors:** Dhafer Kamal, Gowri Sivaramakrishnan, Amr Ashour, Basel M Ebrahim, Maged Morsi

**Affiliations:** 1 Vascular and Endovascular Surgery, King Hamad University Hospital, Bahrain Defence Force Royal Medical Services, Al Sayh, BHR; 2 The Crown Prince Center for Training and Medical Research, Bahrain Defence Force Royal Medical Services, Riffa, BHR; 3 Vascular and Endovascular Surgery, Military Hospital, Bahrain Defence Force Royal Medical Services, Riffa, BHR; 4 General Surgery, King Hamad University Hospital, Bahrain Defence Force Royal Medical Services, Al Sayh, BHR

**Keywords:** postoperative complications, surgical drains, systematic review, vascular surgery, wound healing

## Abstract

Surgical drains are commonly used in vascular surgery to control fluid buildup and reduce postoperative complications; however, their overall benefit remains uncertain due to inconsistent findings and the absence of standardized guidelines. This systematic review aimed to evaluate the role of surgical drains in vascular procedures such as carotid endarterectomy, aortic aneurysm repair, and peripheral arterial bypass, particularly in relation to complications, wound healing, length of hospital stay, and patient recovery. A comprehensive search was conducted across PubMed, Embase, Cochrane, and Google Scholar, following Preferred Reporting Items for Systematic Reviews and Meta-Analyses (PRISMA) guidelines. Studies included randomized controlled trials (RCTs), cohort, and case-control studies that reported on relevant clinical outcomes. Nine studies met the inclusion criteria. The findings revealed mixed results regarding the effectiveness of drains in reducing complications such as hematoma and bleeding, with some benefits noted in specific patient populations. However, an increased risk of surgical site infections was frequently observed with drain use. In certain procedures, such as abdominal aortic aneurysm repair, drains may help prevent the increase in postoperative intra-abdominal pressure, although their influence on overall recovery remains unclear. Given these variable outcomes, the routine use of surgical drains in vascular surgery cannot be universally recommended. Instead, their application should be tailored based on individual patient risk factors and the complexity of the surgical procedure. Additional high-quality studies are needed to develop clear, evidence-based guidelines for their use.

## Introduction and background

Vascular surgery is a specialized field that addresses a wide range of conditions involving the blood vessels, including arterial occlusions, aneurysms, varicose veins, and venous insufficiency. These procedures, which range from simple endovenous treatments to complex revascularization surgeries, are vital for improving circulation and preventing life-threatening complications. However, despite the advances in surgical techniques and perioperative care, patients undergoing vascular surgery remain at significant risk for postoperative complications [1-3]. Among the most common are wound-related issues such as hematomas, seromas, and infections, which may delay wound healing, all of which can adversely affect recovery and prolong hospital stays [2-4]. Surgical drains help prevent fluid buildup, reducing the risk of hematomas, wound infections, and delayed healing [5,6].

Various systematic reviews and meta-analyses in the past examined the use of drainage systems in various surgical contexts and their impact on postoperative outcomes. However, the evidence on the outcomes of drain use across different surgical settings is conflicting. Studies on closed suction drains after mastectomy indicated that drains reduced hospital stays and effluent without affecting seroma formation [7]. In contrast, for obese women undergoing cesarean sections, drains did not significantly impact wound infections or dehiscence but did reduce the risk of hematomas [8]. A meta-analysis of thyroid surgery found that drains did not prevent hematoma formation and were linked to higher infection rates [9]. On the other hand, in cases of aneurysmal subarachnoid hemorrhage, lumbar drains improved clinical outcomes, with lower mortality, fewer vasospasms, and better functional scores compared to no drainage [10].

In vascular surgery practice, some surgeons routinely insert a drain at the end of the procedure (routine drainers). Other vascular surgeons choose to insert a drain in selected cases depending on patient factors, the complexity of the procedure, and the likelihood of bleeding or accumulation of fluids at the operative site (selective drainers). Additionally, the timing of drain removal is debated, with no clear consensus on whether drains should be removed as soon as possible or left in place longer to ensure adequate fluid evacuation [11-13]. Therefore, the absence of standardized guidelines for drain placement leads to variability in clinical practice, contributing to ongoing uncertainty regarding their benefits and risks.

Although the use of surgical drains in vascular surgery is not universally recommended due to potential complications [14], this review seeks to provide clarity on when their use may be appropriate. By synthesizing evidence from various studies, this review aims to identify the scenarios where drains could offer clinical benefits, providing a more nuanced understanding of their role in vascular surgery and helping guide clinical decision-making. Hence, this systematic review aims to critically assess the role of surgical drains in vascular surgery by evaluating the available data on the impact of drains on postoperative complications such as hematoma, infection, and wound healing, as well as their influence on the length of hospital stay, readmission rates, and overall recovery time. By providing a comprehensive overview of the current evidence, this review intends to guide clinical decision-making, improve patient care, and ultimately optimize outcomes in vascular surgery patients.

## Review

Methodology

This systematic review was conducted in accordance with the Preferred Reporting Items for Systematic Reviews and Meta-Analyses (PRISMA) guidelines [15]. This protocol was registered in PROSPERO and is available at: https://www.crd.york.ac.uk/prospero/#recordDetails.

Inclusion and Exclusion Criteria

All clinical and observational studies conducted on humans and published in English were included, with no restrictions on the year of publication. Eligible study designs included published randomized controlled trials (RCTs), cohort studies, case-control studies, and observational studies (prospective or retrospective) that assessed the use of surgical drains in vascular surgeries. Studies such as conference abstracts, book chapters, editorials, case reports, case series, opinion letters, and review articles were excluded. The population included adults or children, with or without comorbidities, undergoing vascular surgical procedures such as, but not limited to, carotid endarterectomy, abdominal aortic aneurysm repair, lower extremity bypass surgeries, and femoral-popliteal bypass surgery. The intervention group comprised studies describing the use of surgical drains - either passive or active - in the postoperative management of any vascular surgical procedure. Comparators included patients undergoing similar procedures without the use of intraoperative drains; however, studies without a comparator group were also considered. Primary outcome measures included the prevention of postoperative complications, specifically hematomas, seromas, wound infections, and other fluid accumulations, as well as the impact on wound healing and overall recovery. Additional outcomes included length of hospital stay, reintervention rates due to complications, and time to recovery.

Search Strategy and Databases

PubMed, EMBASE, Cochrane Library, and Google Scholar were systematically searched for eligible studies. The search strategy combined terms related to vascular surgery procedures (e.g., blood vessel, vascular disease, abdominal surgery, carotid artery, aorta, aortic aneurysm, bypass surgery, vein surgery, arteriovenous fistula, femoro-popliteal bypass, femoro-distal bypass, femoro-tibial bypass, femoro-pedal bypass, popliteal-distal bypass, popliteal-tibial bypass, popliteal aneurysm repair, femoral artery aneurysm repair, embolectomy, carotid endarterectomy, endarterectomy, amputation, limb amputation, aneurysm) with terms related to surgical drainage (e.g., drain, drains, no drain, surgical drains, closed wound drain, Penrose drain, suction drain, open drainage, surgical drainage, vacuum drain). Both Medical Subject Headings (MeSH) and free-text keywords were used where applicable. Boolean operators “OR” and “AND” were used to combine search terms. The search included studies published in English, and filters were applied to include only human studies. Keywords from this search were used alone and in combination to identify other eligible studies. The references of eligible studies were further hand-screened to identify additional qualifying studies.

Data Extraction

Four reviewers conducted an initial screening of several articles and discussed their selections to establish calibration. Following this, the reviewers independently screened the databases for inclusion, resolving any disagreements through discussion. However, if consensus was not achieved, a fifth reviewer intervened, and the study selection was discussed among all authors. The article screening and selection process was facilitated using Rayyan systematic review software [16].

Results

**Figure 1:**
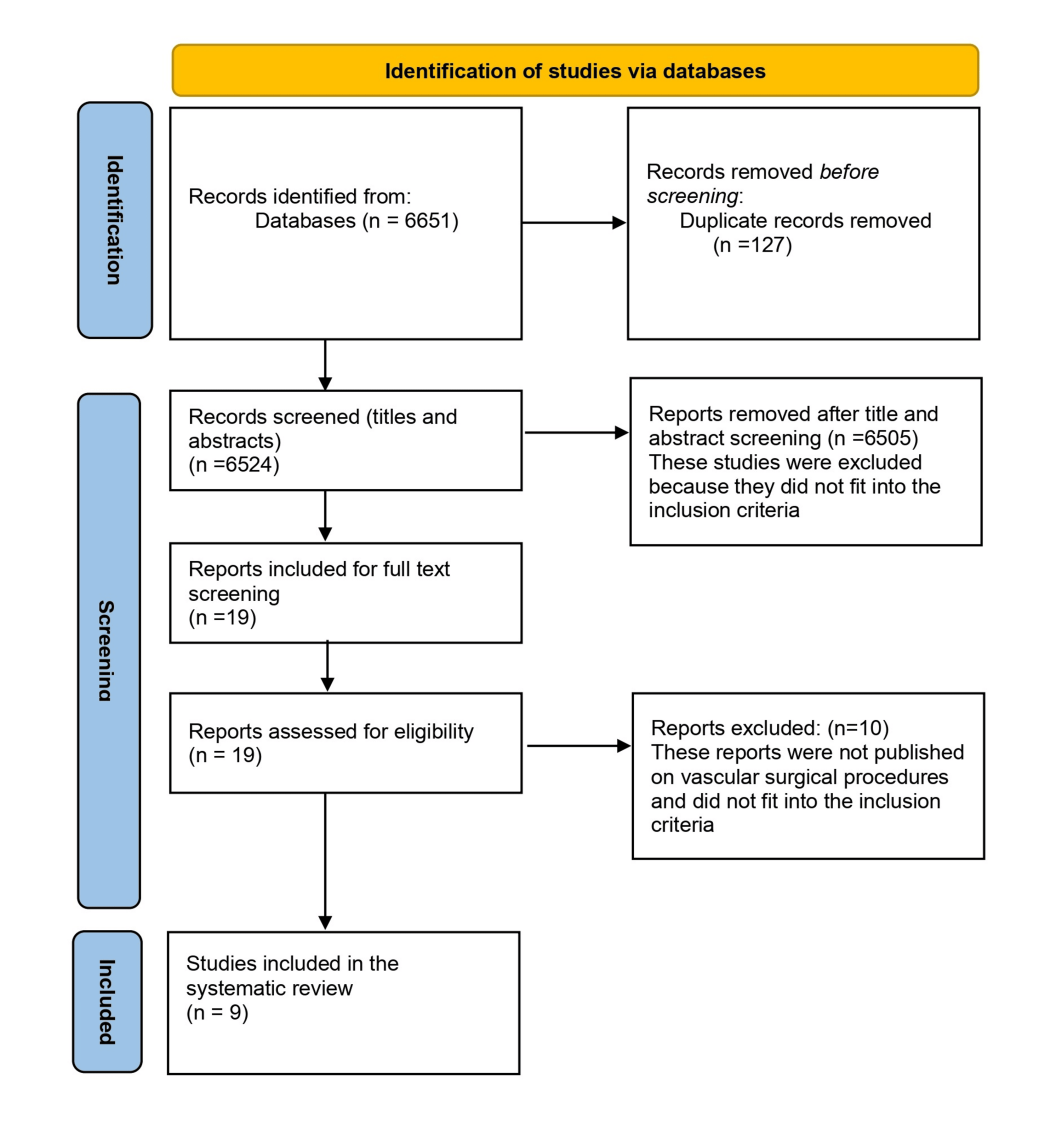
PRISMA flow diagram PRISMA: Preferred Reporting Items for Systematic Reviews and Meta-Analyses

The search yielded 6651 papers initially, which were screened further based on the inclusion criteria. Nine papers were finally included in the systematic review [17-25]. The PRISMA flow diagram is presented in Figure 1.

Table 1 summarizes the key characteristics of the included studies, including study design, sample size, type of vascular surgical procedure, use of drains, and reported outcomes.

**Table 1:** Key characteristics of included studies EVAR: endovascular aneurysm repair; CEA: carotid endarterectomy; OR: operating room; F: French

Study ID	Study design	Population description	Outcome of interest	Results and conclusion from the study
Beard et al. 2001 [17]	Prospective audit	300 consecutive carotid endarterectomies performed for a symptomatic stenosis of the internal carotid artery. They were split into 3 groups of 100 each and clinical practice changes were made according to the outcome	After first 100 - 10F suction drains was discontinued, because they did not prevent 8 wound hematomas. After second 100 - 13 hematomas. Larger 14F drains used for the next set. After third 100 - only 4 hematomas occurred	Large suction drains reduced the incidence of hematoma formation after carotid endarterectomy
Oz et al. 2006 [19]	Prospective study	80 consecutive patients undergoing coronary artery bypass operations. In group I patients (n = 40), we inserted drain during the forearm closing and in group II patients (n = 40), no drain was used	Patients in both groups evaluated for wound site complications such as hematoma, erythema, vascular complications, motor deficit, paresthesia, hand edema, and infection	2 hand edemas, 1 hematoma, 5 paresthesias, 1 infection, and 3 ecchymosis in group I patients. 1 hematoma, 4 paresthesias, 1 infection, and 4 ecchymosis in group II patients. Placing a drain into the forearm has no significant advantages
Manzur-Pineda et al. 2023 [21]	Retrospective study	210 consecutive patients after vascular surgery with common femoral artery exposure. The cohort was divided into 2 groups, groins with drains (n = 59) and without (n = 151) closed suction drains	Outcome was surgical site complications	Closed suction drain group had higher surgical site infection rates (14% vs. 5.6%). The higher risk of infection was not significant after adjustment of confounders such as smoking, diabetes, hyperlipidemia etc. Surgical site infection with drains had lower reintervention rates (37.5%) than those without drains (69%), as well as shorter length of hospital stay. Closed suction drain can be a beneficial adjunct for groin wounds after common femoral artery exposure in patients with comorbidities. Drains decrease the risk of reintervention and length of hospital stay
Sefer 2022 [20]	Retrospective	36 patients who underwent open surgery and endovascular aortic repair treatment for infrarenal abdominal aortic aneurysm. The patients were divided into three groups. Group 1: Patients treated with open surgery and no drains; Group 2: Patients treated with open surgery in whom a drain was placed; Group 3: Patients treated with EVAR	Preoperative and postoperative intra-abdominal pressure measurements	The study found that placing a drain in patients undergoing open surgery is more beneficial to prevent a rise in intra-abdominal pressure
Youssef et al. 2005 [18]	Prospective	70 consecutive patients undergoing carotid endarterectomies and 73 patients who underwent 106 groin dissections were separately and blindly randomized into two groups: group (a) with wound drain and group (b) without wound drain	A duplex scan was carried out postoperatively to document the presence and volume of any wound hematomas	Duplex scan revealed wound hematomas in 8 patients in group (a) and 7 wound hematomas in group (b). In the groin dissection patients, Ultrasound scans showed 21 collections (20%), of these 7 (34%) were in group (a) and 14 (66%) were in group (b). There is no benefit in terms of reduction of the volume of hematomas on wound drainage with the use of drains
Derksen et al. 2009 [22]	Retrospective	A total of 140 patients underwent endarterectomy of the common femoral artery. In 92 of these patients (66%), a 10F percutaneous wound drain was placed at the end of the procedure	Surgical site infection	Infections were reported in 19 out of the 92 patients who had wound drains placed
Jha et al. 2012 [24]	Retrospective	100 consecutive endarterectomies performed by two vascular surgeons. 58 patients in the drain group and 42 in the non-drain group	Bruising and hematoma formation	Bruising and hematoma formation were 15 and 2 in the drain group; compared to 11 and 1 in the non-drain group, respectively. There was no significant difference between the groups.
Smolock et al. 2020 [23]	Retrospective	47,752 patients undergoing carotid endarterectomy. With drain (n = 19,425) and without drain (n = 28,327)	Bleeding, stroke, death, postoperative wound infection, and hospital length of stay	Return to OR for bleeding: No Drain - 236; Drain - 203 Wound infection: No drain - 28; Drain - 14 Drain placement after carotid endarterectomy does not reduce return to the OR for bleeding, nor does it reduce perioperative stroke or death. Drain placement is associated with increased length of stay
Virvilis et al. 2015 [25]	Retrospective	289 carotid endarterectomies were retrospectively analyzed. Closed suction drain was used in 80.2%	Surgical complications	Complications occurred in 193 patients with drains. There is no benefit to temporary vacuum drainage following CEA. There is a higher likelihood of postoperative hematoma in patients with temporary vacuum drainage

Summary of Key Findings in the Included Studies

Study designs and population: The included studies employed both prospective [17-19] and retrospective designs [20-25] to evaluate the impact of wound drains in vascular surgeries. The sample sizes varied significantly across studies. The smallest cohort was 36 patients undergoing abdominal aortic aneurysm repair [20], while Smolock et al. [23] analyzed a large dataset of 47,752 carotid endarterectomy patients. The study population generally included patients undergoing vascular procedures such as carotid endarterectomy and femoral endarterectomy, which entails the removal of an occlusive plaque from the respective vessel and aortic aneurysm repair.

Study outcomes: The primary outcomes assessed across the included studies centered on postoperative complications and the clinical effectiveness of wound drain placement. Hematoma formation was evaluated in four studies [17,18,24,25] to determine whether the use of drains reduced its occurrence. Surgical site infections were investigated in three studies [11-23], comparing infection rates between patients with and without drains. Manzur-Pineda et al. [21] explored the influence of drains on reintervention rates and the duration of hospital stay. One study [20] specifically assessed changes in intra-abdominal pressure in patients undergoing open abdominal aortic aneurysm repair, as elevated intraabdominal pressure can lead to abdominal compartment syndrome, which is a serious postoperative complication affecting organ perfusion and recovery. Additionally, Oz et al. [19] examined a range of wound-related complications, including erythema, vascular complications, motor deficits, paresthesia, edema, and infection in patients who underwent coronary artery bypass surgery. Finally, Smolock et al. [23] investigated whether the placement of drains following carotid endarterectomy reduced the need for return to the operating room due to postoperative bleeding.

Results and Conclusions From These Studies

The findings from these studies present mixed results regarding the benefits and risks of wound drain placement in vascular surgery. Some studies suggest potential advantages, while others highlight increased complications or lack of significant benefits.

Studies Favoring the Use of Drains

Beard et al. [17] found that larger suction drains reduced hematoma formation after carotid endarterectomy. Manzur-Pineda et al. [21] reported that while drains were linked to a higher rate of surgical site infections (14%), they also led to lower reintervention rates (37.5%) and shorter hospital stays, making them beneficial for patients with comorbidities. Sefer [20] demonstrated that drains helped prevent intra-abdominal pressure increases in patients undergoing open abdominal aortic aneurysm surgery.

Studies Against Drain Use

However, several studies argue against the routine use of drains. Oz et al. [19] showed no significant benefit of placing drains in the forearm during coronary artery bypass surgery, as wound complications occurred at similar rates in the group without drains. Youssef et al. [18] found that drains did not reduce wound hematoma volume in carotid endarterectomies or groin dissections. Derksen et al. [22] reported higher infection rates in patients with wound drains, suggesting that drains may increase the risk of infection. Jha et al. [24] observed that bruising and hematoma formation were slightly higher in patients with drains compared to those without. Smolock et al. [23], in a large-scale analysis of 47,752 carotid endarterectomies, found that drain placement did not reduce bleeding, perioperative stroke, or death but was associated with increased hospital length of stay. Similarly, Virvilis et al. [25] concluded that temporary vacuum drainage did not prevent complications and was associated with a higher likelihood of postoperative hematoma. These findings suggest that while drains may offer benefits in specific contexts, their routine use remains debatable due to potential complications such as infections and prolonged hospital stays.

Discussion

This review aims to evaluate the role of surgical drains in vascular surgery, with a focus on understanding whether their use is beneficial in managing postoperative complications, promoting wound healing, and improving recovery outcomes. The results indicate that the effectiveness of wound drains in vascular surgery remains controversial. While some studies suggest benefits in reducing hematoma formation, preventing reintervention, or controlling intra-abdominal pressure, others indicate higher infection rates, increased hospital stays, and no significant reduction in complications [17-25]. The type of surgery and patient comorbidities seem to influence whether drains are beneficial or harmful. Based on these findings, the use of drains in vascular surgery should be carefully evaluated on a case-by-case basis, considering patient risk factors and procedure type.

Patient risk profiles are a key factor in the decision to use drains [26]. Patients with comorbidities like diabetes, obesity, or a history of poor wound healing may be at higher risk for complications such as hematomas or infections that may justify the use of drains. Additionally, older age, anticoagulant therapy, or other anticoagulation-related risks may necessitate drain placement to prevent operative site hematomas and fluid accumulation. Conversely, patients with lower complication risks or those undergoing minimally invasive procedures may not require routine drain placement, supporting a tailored approach.

The complexity of the surgical procedure also plays a crucial role in determining the need for drains [27]. Major arterial reconstructions are more likely to result in postoperative complications, such as fluid accumulation or hematoma formation, making drains a preventive measure. In contrast, simpler procedures with a lower likelihood of complications may not justify the additional risks associated with drains, such as infection or discomfort. Advances in postoperative care have led some to challenge the routine use of drains. Enhanced recovery protocols, improved wound care, and better management of anticoagulation have decreased the incidence of hematomas and infections, potentially reducing the need for drains [28]. In a few studies [19,22,23] included in this review, these improvements may have influenced the outcomes by lowering infection rates regardless of drain use, potentially impacting the perceived benefit of drain placement.

The decision to use a drain, including the choice of type, timing of removal, and consideration of complication risk factors, should be guided by a combination of surgical, patient, and perioperative factors. A thorough risk assessment is essential, considering the nature of the surgery, the patient's medical history, intraoperative findings, and the expected recovery time. Future research into biomarkers or clinical indicators that predict fluid accumulation could provide a more precise framework for decision-making. The authors' opinion based on this study's results and the literature discussed above is that the routine use of drains in vascular surgery should be reconsidered in favor of a more individualized approach. While drains can be beneficial for high-risk patients or complex surgeries, their universal application is not supported by all available evidence. Clear, patient-centered guidelines that account for preoperative, intraoperative, and postoperative factors are needed to ensure judicious use of drains, improving patient outcomes, and minimizing complications. Further studies should identify subgroups of patients who would benefit most from drain placement and develop tailored postoperative care protocols to optimize recovery and minimize risks.

This review, however, has several limitations. The inclusion of only nine studies limits the generalizability of the findings, and the variability in study designs, sample sizes, surgical procedures, and drain size makes direct comparisons difficult. Differences in outcome definitions and follow-up periods may have also influenced the results. Furthermore, the predominance of retrospective studies and potential publication bias reduce the strength of the conclusions.

## Conclusions

This systematic review highlights the nuanced role of surgical drains in vascular surgery. While some evidence suggests that drains may reduce specific postoperative complications such as hematomas in selected patient populations, their routine use remains controversial due to inconsistent findings and the potential for increased surgical site infections. The impact of drains on overall recovery, including wound healing, reintervention rates, and length of hospital stay, also appears variable across procedures and study designs. Given the heterogeneity of available data, the decision to use surgical drains should be individualized, taking into account patient-specific risk factors, the type and extent of surgery, and the surgeon’s clinical judgment. Most procedures may not require prophylactic drain avoiding associated complications. There is a critical need for well-designed, multicenter RCTs to establish evidence-based protocols and stratify the benefit-risk profile of surgical drain use in different vascular surgical contexts. Future studies should also explore the type of drain, duration of use, and cost-effectiveness to provide comprehensive, patient-centered guidelines. Until such data is available, the selective and judicious use of drains remains the most prudent approach.
